# Fatty Acid and Carnitine Metabolism Are Dysregulated in Systemic Sclerosis Patients

**DOI:** 10.3389/fimmu.2020.00822

**Published:** 2020-05-22

**Authors:** A. Ottria, A. T. Hoekstra, M. Zimmermann, M. van der Kroef, N. Vazirpanah, M. Cossu, E. Chouri, M. Rossato, L. Beretta, R. G. Tieland, C. G. K. Wichers, E. Stigter, C. Gulersonmez, F. Bonte-Mineur, C. R. Berkers, T. R. D. J. Radstake, W. Marut

**Affiliations:** ^1^Center for Translational Immunology, University Medical Center Utrecht, Utrecht University, Utrecht, Netherlands; ^2^Department of Rheumatology and Clinical Immunology, University Medical Center Utrecht, Utrecht University, Utrecht, Netherlands; ^3^Biomolecular Mass Spectrometry and Proteomics, Bijvoet Center for Biomolecular Research, Utrecht University, Utrecht, Netherlands; ^4^Referral Center for Systemic Autoimmune Diseases, University of Milan and Fondazione IRCCS Ospedale Maggiore Policlinico, Mangiagalli e Regina Elena, Milan, Italy; ^5^Department of Molecular Cancer Research, Center Molecular Medicine, Oncode Institute, University Medical Center Utrecht, Utrecht, Netherlands; ^6^Department of Rheumatology and Clinical Immunology, Maasstad Hospital, Rotterdam, Netherlands; ^7^Department of Biochemistry and Cell Biology, Faculty of Veterinary Medicine, Utrecht University, Utrecht, Netherlands

**Keywords:** fatty acid oxidation, carnitines, dendritic cells, systemic sclerosis, metabolomics

## Abstract

Systemic sclerosis (SSc) is a rare chronic disease of unknown pathogenesis characterized by fibrosis of the skin and internal organs, vascular alteration, and dysregulation of the immune system. In order to better understand the immune system and its perturbations leading to diseases, the study of the mechanisms regulating cellular metabolism has gained a widespread interest. Here, we have assessed the metabolic status of plasma and dendritic cells (DCs) in patients with SSc. We identified a dysregulated metabolomic signature in carnitine in circulation (plasma) and intracellularly in DCs of SSc patients. In addition, we confirmed carnitine alteration in the circulation of SSc patients in three independent plasma measurements from two different cohorts and identified dysregulation of fatty acids. We hypothesized that fatty acid and carnitine alterations contribute to potentiation of inflammation in SSc. Incubation of healthy and SSc dendritic cells with etoposide, a carnitine transporter inhibitor, inhibited the production of pro-inflammatory cytokines such as IL-6 through inhibition of fatty acid oxidation. These findings shed light on the altered metabolic status of the immune system in SSc patients and opens up for potential novel avenues to reduce inflammation.

## Introduction

Systemic Sclerosis (SSc) is an auto-immune disease with an unknown pathogenesis and unpredictable course. SSc is characterized by vascular lesions, immune cell activation, fibrosis of the skin and internal organs, and loss of the hypodermal fat layer (1). As fat cells are important energy reservoirs, the loss of the fat layer in the fibrotic lesions suggests a role of metabolic changes in SSc. In the last years, metabolomics has shown rapid growth in its application within human health research. The aim of a metabolomics approach is to investigate the complete sets of metabolites within a given sample, in order to achieve a global view of the biological processes within the body (2). Many metabolomics studies have already underlined the importance of metabolism in auto-immune diseases and the metabolomics approach has been applied to identify a fingerprint in diseases such as systemic lupus erythematosus (SLE), Sjögren's syndrome (3), multiple sclerosis, and rheumatoid arthritis (2, 4–6). In SSc, metabolomics pinpoints a distinct metabolic pattern between healthy controls and SSc patients. For instance, a distinct metabolic profile was identified in endothelial cells of SSc patients with pulmonary arterial hypertension (PAH) (7). Other studies have shown a dysregulated fatty acid beta oxidation and amino acid pathway in the urine profile of SSc patients (2, 7–9).

Our group has investigated the role of dendritic cells (DCs) in the pathogenesis of SSc. Previously, we have observed pathological behaviors of DCs in SSc patients, such as a downregulation of RUNX3 expression (10) or overproduction of pro-inflammatory cytokine CXCL4 in plasmacytoid DCs (pDCs) (11).

It has been shown that activated DCs have a different metabolic profile that supports their pro-inflammatory status (12). In the current study, we investigated whether metabolomics assessments in the circulation and intracellularly in DCs of SSc patients, could reveal any metabolic aberrances that might contribute to the pathophysiology of SSc. Therefore, we explored the metabolic profile of the plasma of a large cohort of SSc patients followed by a translational experimental setup. Our results indicate changes in the level of carnitine and subsequently fatty acid metabolism to be altered in the circulation and DCs of patients with SSc. Finally, we demonstrated that etoposide, a drug used in cancer therapy, is able to downregulate inflammation in SSc.

## Methods and Patient Cohort

### Patient Cohort

In compliance with the guidelines of the Declaration of Helsinki and following the approval of the local Institutional Ethical Review Board, peripheral blood was collected after receiving the written informed consent of the patients. The criteria for selecting patients with SSc was performed according to the 2013 American College of Rheumatology (ACR) (13) Classification. Table 1A represents the characteristics of involved patients from Italian discovery cohort from which the plasma was utilized to perform mass spectrometry assessments. Table 1B represents the characteristics of involved patients from Dutch validation cohort from which the immune cells and fibroblasts were utilized to perform *in vitro* assessments. All the blood samples of patients and healthy controls were collected in the morning.

**Table 1A:** Baseline and clinical characteristics of patients with SSc from the discovery cohort, categorized according to the ACR (2013) criteria (The data are presented as mean ± SD or min–max).

**Discovery cohort**	**HC** **(*N* = 7)**	**SSc** **(*N* = 20)**	**ncSSc** **(*N* = 7)**	**lcSSc** **(*N* = 6)**	**dcSSc** **(*N* = 7)**
Age	59 ± 14	57 ± 12	60 ± 9	59 ± 10	52 ± 17
Disease duration (years)	–	13 ± 11	9 ± 5	26 ± 7	7 ± 7
Sex (*n* females)	7 (100%)	17 (85%)	7 (35%)	6 (30%)	4 (20%)
ACR/EULAR score	–	10 ± 2	11 ± 1	12 ± 2	10 ± 2
Raynaud's phenomenon (RP)	–	20 (100%)	7	6	7
Puffy fingers (PF)	–	7 (35%)	7	0	0
Sclerodactyly	–	12 (60%)	0	5	7
Digital ulcers (DU) (anamnestic)	–	5 (25%)	1	3	1
Modified Rodnan skin score (mRSS)	–	4 (0–27)	0	4 (0–6)	12 (5–27)
Telangiectasia	–	10 (50%)	1	5	4
NVC pattern (nailfold video capillaroscopy)	–	9 (45%)	7	–	2
Anti-nucleus antibodies (ANA)	–	20 (100%)	7	6	7
Serum anticentromere (ACA)	–	11 (55%)	6	4	6
Autoantibodies against topoisomerase I (scl70)	–	5 (25%)	0	2	3
RVSP (right ventricular systolic pressure)	–	25.4 ± 5.5	25.3 ± 4.9	25.2 ± 5	24.6 ± 7.3
ILD (interstitial lung disease)	–	4 (20%)	0	1	3
Forced vital capacity (FVC) (% of predicted)	–	106 ± 19	116 ± 18	104 ± 17	96 ± 20
Lung diffusing capacity for carbon monoxide (DLCO) (% of predicted)	–	72 ± 18	71 ± 22	69 ± 12	76 ± 23
Nifedipine	–	19 (95%)	6	6	7
Disease-modifying antirheumatic drugs (DMARDs)	–	5 (25%)	1	1	3

**Table 1B:** Baseline and clinical characteristics of patients with SSc from the validation cohort, categorized according to the ACR (2013) criteria.

**Validation cohort**	**HC** **(*N* = 14)**	**SSc validation** **(*N* = 12)**	**ncSSc** **(*N* = 3)**	**lcSSc** **(*N* = 7)**	**dcSSc** **(*N* = 2)**
Age	42 ± 10	53 ± 9	43 ± 4	56 ± 9	55 ± 3
Disease duration (years)	–	11 ± 10	8 ± 7	14 ± 13	6 ± 9
Sex (*n* females)	12 (86%)	11 (92%)	3 (100%)	7 (100%)	1 (50%)
ACR/EULAR score	–	12 ± 2	12 ± 1	11 ± 2	14 ± 2
Raynaud's phenomenon (RP)	–	12 (100%)	3	7	2
Puffy fingers (PF)	–	7 (50%)	1	3	2
Sclerodactyly	–	5 (42%)	0	3	2
Digital ulcers (DU) (anamnestic)	–	4 (33%)	0	2	2
Modified Rodnan skin score (mRSS)	–	7 (0–19)	0	8 (4–10)	16 (14–19)
Telangiectasia	–	6 (50%)	1	4	1
NVC pattern (nailfold video capillaroscopy)	–	9 (75%)	1	4	2
Anti-nucleus antibodies (ANA)	–	12 (100%)	3	7	2
Serum anticentromere (ACA)	–	3 (25%)	1	2	0
Autoantibodies against topoisomerase I (scl70)	–	6 (50%)	1	4	1
ILD (interstitial lung disease)	–	2 (16%)	0	1	1
Disease-modifying antirheumatic drugs (DMARDs)	–	7 (58%)	0	5	2

*ncSSc, non-cutaneous SSc; lcSSc, limited cutaneous SSc; dcSSc, diffuse cutaneous SSc. (The data are presented as mean ± SD or min–max)*.

### Plasma Collection and Isolation

Venous blood was collected in a 6 mL ACD vacutainer (#364816, BD Biosciences). Blood was further centrifuged for 10 min at 1,500 RPM at room temperature in order to obtain plasma. Next, plasma was aliquoted in sterile Micronics tubes and stored in at −80°C freezer until the experiment date.

## Untargeted Analysis Methods (Italian Discovery Cohort)

### Direct-Infusion High Resolution Mass Spectrometry (DIMS)

Extraction of dried blot spot samples (Ø 3 mm) was performed by ultrasonification for 20 min in 140 μL NSK-AB internal standard solution prepared according to the manufacturer's instructions (Cambridge Isotope Laboratories, Tewksbury, MA, USA). After dilution with 60 μL 0.3% formic acid, the samples were filtered over a 0.2 μm cut-off filter plate (Acroprep, Pall Corporation, Ann Arbor, MI, USA). The samples were collected in a 96-well-plate, sealed to avoid evaporation and subjected to DIMS using an Advion TriVersa NanoMate (Advion, Ithaca, NY, USA) with 5 μm ID chip-based infusion and a Q-Exactive Plus mass spectrometer (Thermo Scientific, Bremen, Germany). Mass spectrometry data were acquired in the scan range of m/z 70–600. The system was operated at 140,000 mass resolution in both positive and negative mode (1.5 min each at 1.6 kV). For high mass accuracy, mass calibration was performed before each experiment and internal lock masses were used (14). Raw data files were converted to mzXML format using MS Convert and processed using an in-house-developed untargeted metabolomics pipeline as well as the HDMB database (accurate mass and isotopic pattern).

### Liquid Chromatography Mass Spectrometry (LC-MS)

A volume of 50 μL of the sample was subjected to water-methanol-chloroform extraction (Folch-method 3). After phase separation by centrifugation, both the aqueous and the organic phase containing all lipids were transferred to clean vials and dried under a gentle stream of nitrogen gas at 40°C.

Prior to analysis, the residue of the aqueous phase was dissolved in 100 μL 10% acetonitrile in ultrapure water. Analysis was conducted with a Thermo Scientific Acella UHPLC system and an Acquity BEH C-8 column (1 ×150 mm, 1.7 μm) kept at 40°C. The column outlet was coupled to a Thermo Scientific Orbitrap XL equipped with an electrospray ion source using both positive and negative ionization. The mass spectrometer was operated in data directed tandem MS mode. The mobile phases consisted of 6.5 mM ammonium carbonate pH 8 (solvent A) and 6.5 mM ammonium carbonate in methanol (solvent B) in the negative mode. For positive mode analysis, the solvents were 0.1% formic acid in ultrapure water and 0.1% formic acid in methanol, respectively. Analysis was started upon injection of 5 μL of sample. A 10 min linear gradient of 0–100% B was started 3 min after the injection of the sample. The system was kept at 100% B for the next 4 min, after which the system returned to its starting situation. The total runtime was 22 min and the flow rate was 150 μL per minute (15).

For lipidomic analysis, the residue of the organic phase was dissolved in 100 μL 80% acetonitrile-20% isopropanol. Analysis was conducted with the system described above using an Acquity BEH C18 column (1 ×100 mm, 1.7 μm) kept at 60°C. The system was operated at a flow rate of 100 μL per minute. The mobile phases consisted of 40% acetonitrile also containing 10 mM ammonium acetate (solvent A), and 10% acetonitrile:90% isopropanol also containing 10 mM ammonium acetate (solvent B) for both negative and positive mode. A 12 min linear gradient of 40–100% B was started after the injection of 5 μL of the sample. The system was kept at 100% B for the next 5 min, after which the system returned to its starting situation. The total runtime was 20 min.

For both the analysis of polar and non-polar (lipid) metabolites, the acquired MS-data was processed using MZMine 2 open source software 4 and searched against available databases.

## Targeted Analysis Methods (Dutch Validation Cohort)

### Acylcarnitine Analysis

For each analysis, a volume of 50 μL NSK-B internal standard solution was mixed with 50 μL of plasma and 300 μL acetonitrile, according to the manufacturer's instructions. The sample was centrifuged at 4° at 14,000 × g for 5 min, and the supernatant was transferred to a gas chromatography (GC) vial and evaporated to dryness at 40°C under a gentle stream of nitrogen. A volume of 100 μL freshly prepared butylation reagent was added to the residue. The vial was then vortexed, incubated at 60°C for 15 min, and evaporated to dryness at 40°C under a gentle stream of nitrogen. The residue was dissolved in 100 μL acetonitrile and subjected to analysis. Standards and quality control samples were prepared similarly (16).

Samples were analyzed on a Waters XEVO Triple Quadrupole mass spectrometer using an Acquity UPLC system for sample delivery (Waters, Milford, MA USA). For the analysis of the samples, 5 μL of the derivatized sample was injected via the systems bypass via a restrictor into an 400 μL per minute acetonitrile flow. The MS system was operated in the positive ionization mode using MRM scanning (parent–daughter masses) with analyte dependent collision energy for acetyl-carnitine identification and quantification.

### Fatty Acid Analysis

Arachidonic acid-D8 in methanol (10 μL) was added to a sample (20 μL) and subjected to water-methanol-chloroform extraction (Folch-method). After phase separation by centrifugation, the organic phase containing all lipids was removed from the vial and dried under a gentle stream of nitrogen gas at 40°C. The residue was dissolved in chloroform and subjected to clean-up by solid phase extraction (SPE) on amino-silica columns (17). After SPE, the fraction containing the FFA was dried under a stream of nitrogen at 37°C. The residue was dissolved in 100 μL acetonitrile and subjected to LC-MS analysis using an Acella UHPLC coupled to a LTQ-Orbitrap XL MS. The LC was equipped with an Acquity BEH-C18 column (2.1 ×5 cm, 1.7 μm) and guard column. Fatty acids were separated by means of a 20 min 10–95% acetonitrile gradient in 0.1% acetic acid at 300 μL per minutes and 60°C. FFA identification and response quantification was performed using retention time (±0.1 min) and ion m/z-values ( ≤ 5 ppm).

### Monocyte-Derived DCs (moDCs) Differentiation and Stimulation

Peripheral blood mononuclear cells (PBMCs) from HC and SSc patients were isolated by a Ficoll (GE Healthcare) gradient. Monocytes were isolated using an autoMACS Pro Separator (Miltenyi Biotec) according the manufacturer's instructions. Purity was routinely assessed by flow cytometry and above 94%. Monocytes were seeded at a final concentration of 1 million per ml and cultured in RPMI-GlutaMAX (Thermo Fisher Scientific) for 6 days. Medium was supplemented with 10% FBS (Biowest), 10,000 I.E. penicillin-streptomycin (Thermo Fisher Scientific), recombinant human granulocyte-macrophage colony-stimulating factor (GM-CSF, 800 IU/mL; R&D Systems), and recombinant human interleukin-4 (IL-4, 500 IU/mL; R&D Systems), as previously described (18).

### Mass Spectrometry on moDCs

After 3 and 24 h, moDCs were harvested and centrifuged for 5 min at 1,000 G. Medium samples were collected and the cell pellet was washed with ice-cold PBS. Metabolites were extracted by adding 50 μl of ice-cold MS lysis buffer [methanol/acetonitrile/ULC/MS grade water (2:2:1)] to the cell pellet. Samples were shaken for 10 min at 4°C and centrifuged at 14,000 G for 15 min, after which the supernatants were collected for LC-MS analysis. LC-MS analysis was performed on an Exactive mass spectrometer (Thermo Scientific) coupled to a Dionex Ultimate 3000 autosampler and pump (Thermo Scientific). The MS operated in polarity-switching mode with spray voltages of 4.5 and −3.5 kV. Metabolites were separated using a Sequant ZIC-pHILIC column (2.1 ×150 mm, 5 μm, guard column 2.1 ×20 mm, 5 μm; Merck) using a linear gradient of acetonitrile and eluent A [20 mM (NH_4_)_2_CO_3_, 0.1% NH_4_OH in ULC/MS grade water (Biosolve)]. The flow rate was set at 150 μl/min. Metabolites were identified and quantified using Lcquan software (Thermo Scientific) on the basis of exact mass within 10 ppm and further validated by concordance with retention times of standards. Peak intensities were normalized based on total intensities per time point.

### Fibroblast Culture and Stimulation

Dermal fibroblasts obtained from healthy controls and SSc patients were cultured in DMEM medium (Thermo Fisher Scientific) 10% FBS (Biowest) and 10,000 I.E. penicillin-streptomycin (Thermo Fisher Scientific) at 37°C and 5% CO_2_. The day prior to the experiment, fibroblasts were seeded at a final concentration of 7,000 cells per mL. Fibroblasts were starved prior to be stimulated with TGFβ2 (R&D cat#302-B2-002/CF). On the day of the experiment the medium was refreshed and supplemented with etoposide at a final concentration of 10 or 1 nM.

### RT-PCR and Quantitative (q)PCR

RNA from fibroblasts was isolated using the Allprep Universal miRNA/RNA/DNA kit from Qiagen. Total RNA was reverse transcribed using SuperScript™ IV kit by Invitrogen. Duplicate PCR reactions were performed using SYBR greenselect Master Mix (Applied Biosciences) and measured with a Quantstudio 12K flex Real-Time PCR detection system. cDNA was amplified using specific primers (RPL13αfw: CCTGGAGGAGAAGAGGAAAGAGA rw: TTGAGGACCTCTGTGTATTTGTCAA, TAGLN fw: CTCATGCCATAGGAAGGACC rw: GTCCGAACCCAGACACAAGT, α*SMA* fw: CCGACCGAATGCAGAAGGA rw: ACAGAGTATTTGCGCTCCGAA, *Col1a1* fw: CCAGAAGAACTGGTACATCAGCA rw: CGCCATACTCGAACTGGAAT, *CTGF* fw: AGCTCGGTATGTCTTCATGCTGGT rw: TTGCGAAGCTGACCTGGAAGAGAA). Relative levels of gene expression were calculated by normalizing to *RPL13*α housekeeping gene. Fold changes (FC) of mRNA were calculated by using the formula 2^−ΔΔ*Ct*^.

### Interleukin 6 Quantification Using ELISA

Interleukin (IL-)6 was quantified in cell-free supernatants using an ELISA-based PeliKine compact™ human IL-6 kit (Sanquin, Amsterdam, The Netherlands), and this was performed following the manufacturer's instructions.

### Viability Assessment on moDCs

Annexin V-7AAD staining was used to assess cell death. Cells were stained with Annexin V (1:100 dilution, BD Pharmingen) and 7AAD (1:100 dilution, BD Pharmingen) and measured using a FACS Canto Flow Cytometer. The data were further analyzed with BD FACS DIVA software.

### Statistical Analysis

Statistical analysis was performed via website platformed based pipeline tool: https://www.metaboanalyst.ca. Distance between samples was measured with Pearson. Where appropriate, a Mann–Whitney test or Paired *t*-test was assessed using Graph Pad Prism 8.0 Software. *P*-values smaller than 0.05 were considered as statistically significant.

## Results

### Different Metabolic Pattern Between HC and SSc

**Figure 1:**
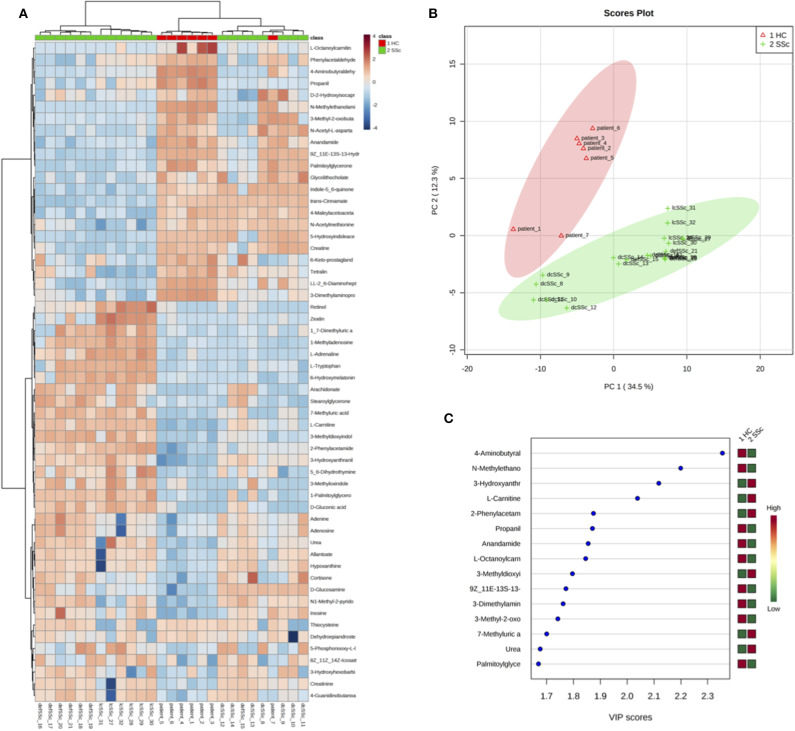
Differentially abundant compounds in plasma from HC and SSc patients. **(A)** Heatmap of the 56 significantly different compounds identified in plasma of HC and SSc patients. **(B)** Partial Least Squares Discriminant Analysis of plasma samples from HC and SSc patients. **(C)** Variable Importance in Projection score obtained from the Partial Least Squares Discriminant Analysis.

After screening the spectra obtained from the plasma of 27 individuals, a total of 157 compounds were identified. Using the online platform from Metaboanalist.ca, we performed *t*-test statistical analyses and a total of 56 compounds were identified as having a different level in plasma from SSc patients compared to HC (Table 2). Next, a heatmap of the 56 identified metabolites was generated and is shown in Figure 1A. Briefly, the heatmap reveals different levels of metabolites involved in processes such as fatty acid oxidation (FAO) [L-carnitine and fatty acid derived esters of carnitine (acyl-carnitines)] or kidney function (such as urea and creatinine) were observed in patients compared to HC. With the same tool, we generated a principal component analysis (PCA) and a Partial Least Squares Discriminant Analysis (PLS-DA) (Supplementary Figure 1, Figure 1). The result of the PCA and PLS-DA revealed clear separation between HC and SSc patients based on the levels of metabolites detected in plasma (Figure 1B). In order to identify the variable most efficient in separating the HC from the SSc patients, the variable importance in projection (VIP) score was generated. The results are shown in Figure 1C. The VIP score showed that L-carnitine and acyl-carnitines were relevant for the distinction between HC and SSc patients.

**Table 2:** *T*-test results of the features with different level in plasma from SSc compared to HC.

**Compound**	***t*-stat**	***p-*value**	**–LOG10(*p*)**	**FDR**
4-Aminobutyraldehyde	20.626	1.71E−16	15.768	2.70E−14
N-Methylethanolamine phosphate	15.732	4.30E−13	12.367	3.39E−11
Anandamide	8.5994	6.34E−09	8.1982	2.55E−07
L-Carnitine	−9.4886	6.46E−09	8.1895	2.55E−07
9Z_11E-13S-13-Hydroperoxyoctadeca-9_11-dienoic acid	8.6604	4.88E−08	7.3119	1.33E−06
3-Methyldioxyindole	−7.7001	5.05E−08	7.2965	1.33E−06
7-Methyluric acid	−7.5374	1.07E−07	6.9727	2.40E−06
Arachidonate	−6.9706	3.04E−07	6.5172	6.00E−06
3-Methyl-2-oxobutanoic acid	6.9281	4.30E−07	6.3665	7.55E−06
Urea	−6.6387	6.69E−07	6.1745	1.06E−05
Phenylacetaldehyde	6.7867	7.84E−07	6.1059	1.13E−05
5-Hydroxyindoleacetate	5.8472	1.08E−05	4.9657	0.00014249
Creatine	5.6352	1.19E−05	4.9227	0.00014522
Cortisone	−5.5075	2.19E−05	4.6593	0.00024732
Palmitoylglycerone phosphate	5.6637	2.56E−05	4.5917	0.00026969
Allantoate	−5.145	2.80E−05	4.553	0.00027639
N-Acetyl-L-aspartate	4.938	4.39E−05	4.3579	0.00040771
2-Phenylacetamide	−6.016	7.02E−05	4.154	0.00061579
LL-2_6-Diaminoheptanedioate	5.233	0.00010047	3.998	0.00083545
Tetralin	4.5971	0.0001385	3.8585	0.0010942
Trans-Cinnamate	4.5062	0.00017441	3.7584	0.0013122
4-Maleylacetoacetate	4.3634	0.00019949	3.7001	0.0014327
L-Tryptophan	−4.2562	0.00026453	3.5775	0.0018172
1-Palmitoylglycerol 3-phosphate	−4.2311	0.00027612	3.5589	0.0018178
1-Methyladenosine	−4.1094	0.00040482	3.3927	0.0025585
3-Hydroxyanthranilate	−6.0887	0.00042674	3.3698	0.0025933
L-Adrenaline	−3.8816	0.0006982	3.156	0.0040857
3-Dimethylaminopropyl benzoate	4.7859	0.00078949	3.1027	0.004455
Propanil	5.0085	0.00082285	3.0847	0.0044831
Stearoylglycerone phosphate	−3.7659	0.00091976	3.0363	0.0048441
Hypoxanthine	−3.736	0.00098251	3.0077	0.0050076
D-2-Hydroxyisocaproate	3.722	0.0011592	2.9359	0.0054985
6-Hydroxymelatonin	−3.6648	0.0011673	2.9328	0.0054985
Indole-5_6-quinone	3.6726	0.0011832	2.9269	0.0054985
N-Acetylmethionine	3.6107	0.0013535	2.8685	0.0059476
N1-Methyl-2-pyridone-5-carboxamide	−4.2214	0.0013551	2.868	0.0059476
D-Gluconic acid	−3.8235	0.0014258	2.8459	0.0060886
Adenine	−3.787	0.0018606	2.7303	0.0077362
Creatinine	−3.5091	0.0019132	2.7182	0.0077509
Retinol	−3.3348	0.0029065	2.5366	0.011481
5_6-Dihydrothymine	−3.4016	0.0031036	2.5081	0.01196
5-Phosphonooxy-L-lysine	−3.2559	0.0033209	2.4787	0.012493
3-Methyloxindole	−3.2515	0.0042893	2.3676	0.015641
Zeatin	−3.1636	0.0043557	2.3609	0.015641
Adenosine	−3.0758	0.0063492	2.1973	0.022293
Thiocysteine	3.0117	0.0069451	2.1583	0.023855
Inosine	−3.5235	0.0080192	2.0959	0.026958
1_7-Dimethyluric acid	−2.8674	0.0083164	2.0801	0.027375
Glycolithocholate	2.9311	0.0092378	2.0344	0.029787
D-Glucosamine	−3.2818	0.010531	1.9775	0.033279
3-Hydroxyhexobarbital	−2.8883	0.010945	1.9608	0.033907
6-Keto-prostaglandin F1alpha	3.017	0.012337	1.9088	0.036936
Dehydroepiandrosterone sulfate	2.7262	0.01239	1.9069	0.036936
L-Octanoylcarnitine	3.3887	0.0138	1.8601	0.040378
4-Guanidinobutanoate	−2.7708	0.014298	1.8447	0.041073
8Z_11Z_14Z-Icosatrienoic acid	−2.9042	0.016084	1.7936	0.045379

*ncSSc, non-cutaneous SSc; lcSSc, limited cutaneous SSc; dcSSc, diffuse cutaneous SSc*.

### SSc Patients Have Dysregulation of Fatty Acids and Carnitines

**Figure 2:**
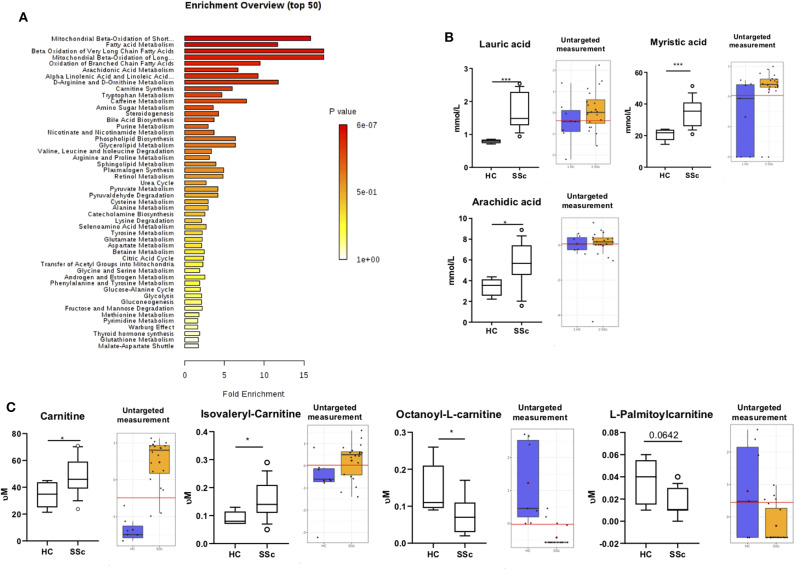
Fatty acids and Carnitine are altered in SSc. **(A)** Summary Plot for Over Representation Analysis of differentially abundant compounds in plasma from SSc patients and HC. **(B)** On the left, quantification of lauric acid, myristic acid, and arachidic acid with targeted approach. On the right quantification with untargeted approach. **(C)** On the left, quantification of L-carnitine, Isovaleryl-carnitine, Octanoyl-carnitine, and Palmitoyl-carnitine with targeted approach. On the right, quantification with untargeted approach (Box are represented as the 10–90th percentile. *P ≤ 0.05, **P ≤ 0.01, ***P ≤ 0.001, and ****P ≤ 0.0001). Red lines represent optimal cutoff. Red dots represent the mean concentration of each group.

Next, we performed the quantitative enrichment analysis script from Metaboanalist.ca pipeline (Figure 2A). We found multiple metabolic processes to be altered in the plasma of SSc patients as compared to HC, involving fatty acid (FA) and L-carnitine, such as mitochondrial beta oxidation of short chain saturated FA, FA metabolism, beta oxidation of very long chain FA, and carnitine synthesis pathways, to be altered in the plasma of SSc patients as compared to HC. These observations, suggested an inter relation of FA and carnitine involvement in SSc patient's metabolic profile.

To confirm our observation, we performed targeted analysis focusing specifically on FA and carnitine. We observed an increase of lauric acid (*P* = 0.0001), myristic acid (*P* = 0.0009), and arachidic acid (*P* = 0.015) (Figure 2B) in the plasma of SSc patients when compared to HC.

**Figure 3:**
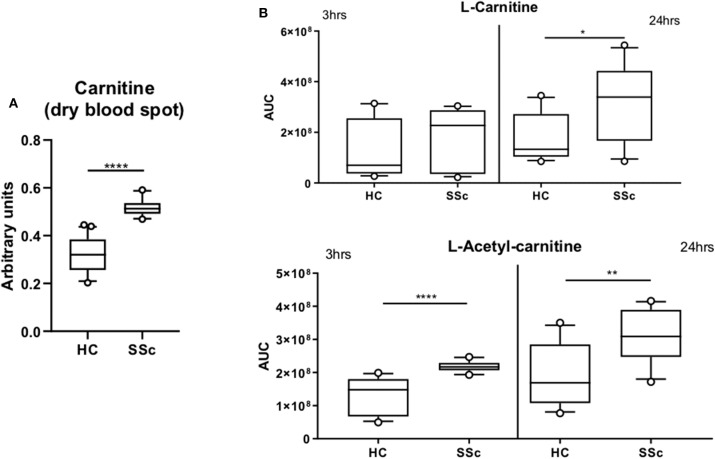
Carnitine is increased in SSc. **(A)** Quantification of L-carnitine in dry blood spot measurement. **(B)** Quantification of L-carnitine and L-Acetyl-carnitine in four healthy controls and four SSc moDCs executed in triplicate and incubated for 3 or 24 h (AUC, Arbitrary unit count; boxes are represented as 10–90%. *P ≤ 0.05, **P ≤ 0.01, ***P ≤ 0.001, and ****P ≤ 0.0001).

Furthermore, we found an increase of the carnitine (*P* = 0.025) and Isovaleryl-carnitine (*P* = 0.03) and a decrease of Octanoyl-carnitine (*P* = 0.04) and Palmitoyl-carnitine (*P* = 0.06; Figure 2C) in the plasma of SSc patients. These findings are in line with our previous observations using the untargeted panel, further suggesting the presence of an imbalance of FA and carnitines in SSc. To further confirm the alteration of carnitine in the circulation of SSc patients, we measured carnitines using dry blood spot, where we confirmed increased level of carnitines in plasma of SSc patients (*P* <0.0001). Taken together, we confirmed carnitine alteration in pooled SSc patients using three techniques in independent measurements (Figures 2C, 3A).

### Carnitine Alterations in the Immune Cells From SSc Patients

Furthermore, we investigated whether carnitine alterations were also present at the cellular level in SSc patients. Since the role of dendritic cells in SSc pathogenesis is our main focus, we measured the basal level of carnitine in monocytes derived dendritic cells (moDCs) at two different time points (3 and 24 h). We observed an increase in L-carnitine after 24 h incubation (*P* = 0.023) and L-acetyl-carnitine (*P* <0.0001 at 3 h and *P* = 0.0086 at 24 h) in SSc moDCs when compared to HC moDCs (Figure 3B). These results further highlight the potential importance of carnitine in the altered metabolism of SSc patients.

### Fatty Acids and Carnitine Levels in SSc Disease Subsets

In order to gain more insight on FA and carnitine levels per disease subset we performed Mann-Whitney *t*-test per subgroup of ncSSc, lcSSc, and dcSSc in plasma and dry bloodspots samples. The comparison was made between the subsets and HC (Table 3, Figures 2B,C).

**Table 3:** Statistical comparisons of fatty acid and carnitine levels in plasma and dry blood spots between ncSSc, lcSSc, and dcSSc vs. healthy controls.

**Plasma FA**	**ncSSc** **(*n* = 3)**	**lcSSc** **(*n* = 7)**	**dcSS** **(*n* = 2)**
Lauric-acid	0.0079	0.0043	0.016
Myristic-acid	0.0079	0.0043	0.11
Arachidic-acid	0.016	0.05	0.29
Dry blood spot	(*n* = 6)	(*n* = 6)	(*n* = 6)
Carnitine	<0.0001	<0.0001	<0.0001
Plasma carnitine	(*n* = 7)	(*n* = 6)	(*n* = 7)
Carnitine	0.2222	0.0932	0.0159
Isovaleryl-carnitine	0.553	0.0215	0.0159
Octanoyl-carnitine	0.0556	0.1111	0.2857
L-palmitoyl-carnitine	0.1429	0.0671	0.4683

### Dysregulation of the Fatty Acid Oxidation and Carnitines in SSc Patients Promotes Inflammation and Fibrosis

**Figure 4:**
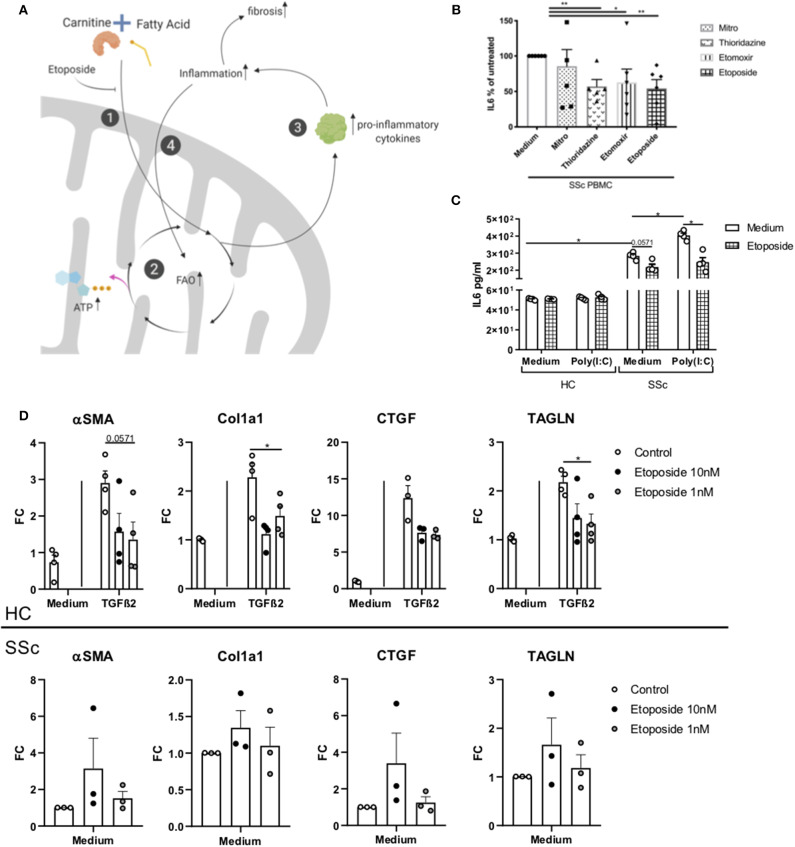
Effect of Etoposide on inflammation and fibrotic genes. **(A)** Schematic representation of the FAO and etoposide effect. (1) Carnitine and fatty acid combine to form an acyl-carnitine. This is transported inside the mitochondria. Etoposide, blocking the intake of carnitine limits the formation of acyl-carnitine in the cell. (2) The acyl-carnitine combines with CoA in fatty acyl-CoA, this enters the FAO to generate ATP. (3) FAO can promote the release of pro-inflammatory cytokines, (4) promoting inflammation. Inflammation promotes fibrosis, and (5) promotes FAO, generating a vicious circle. Created with Biorender. **(B)** IL6 quantification in PBMCs from six SSc patients exposed to mildronate (1 mmol/L), thioridazine (10 μM), etomoxir (5 μM), and etoposide (10 nM), expressed as a percentage. **(C)** IL6 quantification in moDCs from four HC and four SSc patients exposed to etoposide. **(D)** PCR quantification of *TAGLN*, α*SMA, Col1*, and *CTGF* genes in four HC fibroblasts stimulated with TGFβ and three unstimulated SSc fibroblasts treated with etoposide at 10 or 1 mM (Bars are represented as mean ± SEM. *P ≤ 0.05). **P ≤ 0.01.

We hypothesize that the alteration in FA and carnitines observed in SSc patients are a manifestation of dysregulated FAO. Alternation in FAO leads to increase production of pro-inflammatory cytokines and inflammation (19, 20). Inflammation is known to induce fibrosis and therefore, promotes a vicious circle which further endorses the FAO (21) (Figure 4A).

Inflammation and fibrosis are two main features observed in SSc patients. To test the hypothesis that the FAO is dysregulated in SSc, leading to a vicious circle where inflammation and FAO induce each other, we investigated the role of different carnitine inhibitors (etoposide, thioridazine, mildronate, and etomoxir) by testing the production of pro-inflammatory cytokine in immune cells. Etoposide is a molecule with an inhibitory effect on the organic cation/carnitine transporter (OCTN2), while thioridazine inhibits peroxisomal oxidation of lipids (22, 23), and mildronate is an inhibitor of the mitochondrial carnitine/acyl-carnitine transporter (24). To study the effect of carnitine inhibitors on the production of pro-inflammatory cytokines, we used SSc and HC PBMCs. Since PBMCs from SSc patients are known to spontaneously produce pro-inflammatory cytokines, such as interleukin (IL-) 6 (25), we used IL-6 as readout of the cytokine production. We observed that etoposide (10 nM), etomoxir (5 μM) and thioridazine (10 μM), but not mildronate (1 mmol/L), was able to significantly reduce the production of IL-6 (*P* = 0.0022 for etoposide and thioridazine and *P* = 0.0476 for etomoxir), in PMBCs from SSc patients (Figure 4B). The least variation between the donors were observed with etoposide, and further experiments were therefore performed using etoposide only. Furthermore, etoposide, thioridazine, mildronate, and etomoxir did not affect cell viability (Supplementary Figure 2).

### Etoposide Downregulates Inflammatory Response in SSc moDCs and Suppresses Fibrotic Gene Expression in Healthy and SSc Fibroblasts

Next, moDCs generated from HC and SSc were stimulated with Poly(I:C) (TLR3 ligand) and cultured in the presence of etoposide for 24 h. We observed no induction of IL-6 in healthy moDCs exposed to TLR3 and/or etoposide. Interestingly, we found a reduction of IL-6 in SSc moDCs cultured in the presence of etoposide, both, with and without TLR3 stimulation (respectively, *P* = 0.057 and 0.028; Figure 4C). Furthermore, we investigated the effect of etoposide on fibrotic genes in healthy fibroblasts stimulated with TGFβ2 and unstimulated fibroblasts from SSc patients. We measured the expression of transgelin (TAGLN), actin alpha 2 smooth muscle (αSMA), collagen 1a (Col1a) and connective tissue grow factor (CTGF). We observed that, in healthy fibroblasts stimulated with TGFβ, TAGLN (*P* = 0.029), and Col1a (*P* = 0.028) were reduced, while αSMA (*P* = 0.057) and CTGF (*P* = 0.061) showed a trend of reduction (Figure 4D). Unstimulated fibroblasts from SSc patients showed no reduction of TAGLN, αSMA, Col1a, and CTGF (Figure 4D). Our data suggests that etoposide have an anti-inflammatory effect on SSc moDCs and an anti-fibrotic effect on healthy fibroblasts stimulated with TGFβ, but not in unstimulated SSc fibroblasts. These results open a new avenue to exploit fatty acid inhibition. Further studies are required in order to delineate the role of etoposide in the mechanism of inflammation involved in SSc given its potential anti-fibrotic effect shown on HC stimulated fibroblasts.

## Discussion

The aim of our study was to identify and explore the potential role of circulatory and intracellular metabolites in the development of inflammation in SSc patients.

We observed an altered FA and carnitines profile in both the blood and immune cells (plasma and DCs) of SSc patients. Carnitine is a molecule with a structure similar to amino acids that is present in every mammalian species. Carnitine can be both taken up by food or being endogenously produced (26). Carnitine plays an important role in cellular energy metabolism. In fact, carnitine transports fatty acids (as acyl-carnitine) into the mitochondria in order to allow FA to be oxidized (26). Therefore, carnitine and FA, via the FAO, are the fundamental key players for the energy metabolism of cells. It has been shown that an altered FA metabolism is reflected by defective composition of acyl-carnitines (27). In line with this, we observed alteration in acyl-carnitines, such as Isovaleryl-, Palmitoyl-, and Octanoyl- carnitine. We speculate that the increase of Isovaleryl-carnitine could derive from an excess of amino acids consumption in order to generate ATP. Furthermore, the reduction observed in Palmitoyl-carnitine levels, a factor known to potentially facilitate the transfer of long-chain FA from cytoplasm into mitochondria during the oxidation of fatty acids (28, 29), could imply a dysfunction in the transport of long chain FA inside the mitochondria. This could lead to an imbalance in long chain FA inside of the mitochondria, which could potentially be responsible for the observed low level of Octanoyl-carnitine (a long chain FA derived acyl-carnitine). Taken together, we speculate that our observation of increased Isovaleryl-carnitine could depend on the use by cells of amino acids in order to generate ATP and potentially be induced by a dysfunction in the oxidation of long chain FA.

Briefly, our observations signify an altered energy metabolism in immune cells and plasma of SSc patients that is reflected by a dysregulated FA and carnitine profile, including acyl-carnitines.

Immune cells with altered metabolism lead to aberrant immune response (30, 31). In macrophages, increased FA metabolism triggers a disturbed immune response, difficulties in adapting to the surrounding environment, and shifting toward pro-fibrotic M2 phenotype (32, 33). Furthermore, studies on T helper cells showed that an altered FA metabolism and a disturbed (micro)environment surrounding the naïve T helper cells, predict the metabolic programming of the cells. Inhibition of FAO shifts the T helper cell differentiation more toward the pro-inflammatory T helper 17 phenotype (34). Therefore, in SSc, an increase of the FA metabolic profile of the immune cells, might worsen the disease prognosis by priming a pro-inflammatory programmed immune system. Moreover, dysfunction of FAO was found to play an important role in the direct induction of renal fibrosis development (35). Both inflammation and FAO amplifies the production of pro-inflammatory cytokines (20, 21, 36), and, thereby, they both substantiates the vicious circle of chronic inflammation and fibrosis.

To better understand the significance of FAO in SSc, we blocked the cellular carnitine intake by inhibiting the OCTN2 transporter using etoposide. Etoposide is a well-known drug available for cancer treatment as i.e. prostate cancer, small cell lung carcinoma, and leukemia (37, 38). Etoposide inhibits carnitine transporter OCTN2 (39), which prevents FAO and subsequently might potentiate the downregulation of pro-inflammatory immune system. In addition, etoposide has been shown to inhibit topoisomerase II that might impact the experimental results in proliferating cells (40). As monocyte-derived dendritic cells do not divide in culture and the incubation time with the inhibitor was short, the expected impact on the experimental results is unlikely to occur.

Furthermore, etoposide is suggested to be used in combination with corticosteroids or other DMARDS in treatment of systemic inflammation in Still's disease (41), which is a rare disease with rheumatoid arthritis-like hallmarks. In our studies, etoposide showed anti-inflammatory properties on DCs from SSc and anti-fibrotic properties on TGF-β stimulated fibroblasts from healthy donors but not in unstimulated SSc fibroblast. This observation could be explained by potential epigenetic alterations since SSc fibroblast were *in vivo* chronically exposed to a pro-inflammatory environment. It is indeed known that chronic exposure to stress can induce epigenetic modifications in cells. For instance, cardiac fibroblasts exposed to hypoxia (42) were shown to epigenetically acquire an hyper-reactive fibrotic phenotype. The pro-fibrotic phenotype is acquired after global DNA hypermethylation induced by continuous expression of hypoxia inducible factor 1α (42). Interestingly, hypoxia, together with a dysregulated and chronic immune activation, is the key feature of fibrogenesis in SSc (43). Therefore, it is likely that the overexpression of pro-fibrotic genes in SSc fibroblasts might be eventuated by hypoxia inflicted epigenetic modifications present in patients with SSc. However, further studies need to be performed to support this hypothesis.

One limitation of this study is the absence of information regarding the fasting status of the participants. However, fasting status is not expected to have much influence on the study results since all participants of each cohort (healthy and SSc patients) were included in a similar fashion, and the blood was drawn at approximately the same time.

In conclusion, targeted suppression of the FAO metabolism could be helpful to inhibit inflammation in SSc and therefore might offer a novel therapeutic target. While the literature on FAO and carnitine in SSc is rather poor, we believe that our results provide an intriguing and robust foundation to further elucidate the pathogenic mechanisms taking place in SSc immune dysregulation.

## Data Availability Statement

All datasets generated for this study are included in the article/Supplementary Material.

## Ethics Statement

The studies involving human participants were conducted in compliance with the guidelines of the Declaration of Helsinki and following the approval of the local Institutional Ethical Review Board from UMC Utrecht, Maasstad Ziekenhuis, and Ospedale Maggiore Policlinico, Mangiagalli e Regina Elena. The patients/participants provided their written informed consent to participate in this study.

## Author Contributions

All authors approved the final version after being involved in drafting and revising the article for important intellectual content. AO and WM had full access to the data and take responsibility for the accuracy of the performed analysis and the integrity of the data. AO, TR, and WM were involved in design of the study. Execution and analysis of the results was performed by AO, AH, MZ, MK, CW, NV, MC, EC, MR, LB, RT, ES, and CG, and they were all involved in performing experiments. AO and MC were involved in selection of the patients. TR, LB, and FB-M were involved in inclusion of SSc patients. All authors contributed to the review of the manuscript.

## Conflict of Interest

The authors declare that the research was conducted in the absence of any commercial or financial relationships that could be construed as a potential conflict of interest.
