# Supplementary material for: Fatty Acid and Carnitine Metabolism Are Dysregulated in Systemic Sclerosis Patients
Source: Front Immunol. 2020 May 22;11:822. doi: 10.3389/fimmu.2020.00822 (PMC7256194; doi:10.3389/fimmu.2020.00822)
Supplement: Supplementary file 1 [file Data_Sheet_1.PDF]

Supplementary Material

1.1 Supplementary Figures

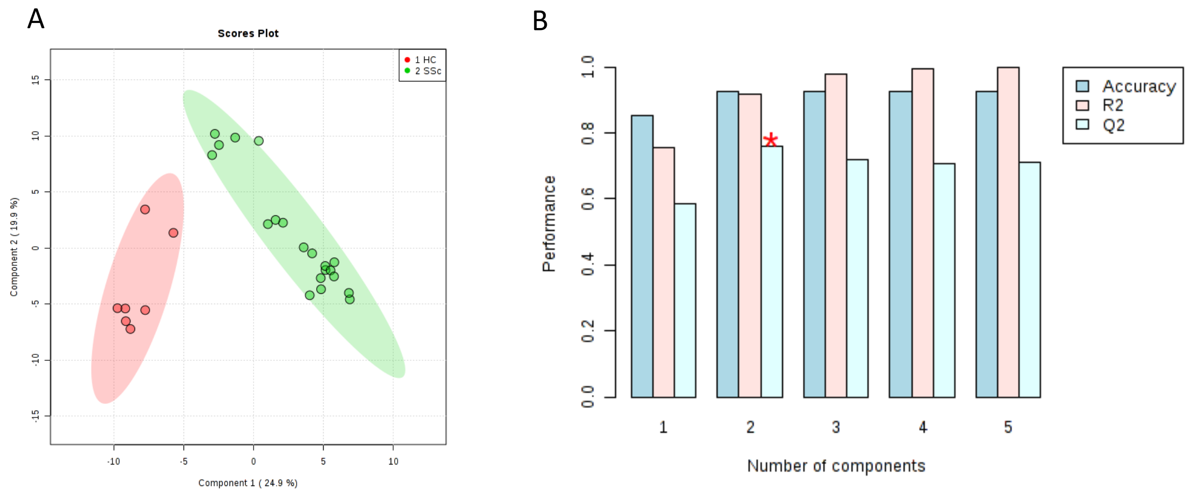

Supplementary Figure 1. A) Principal Component Analysis (PCA) of plasma samples from HC and SSc patients. **B)** PLS-DA model evaluation R2Q2 values. The variability of ~ 5% were observed when the internal standard was repeatedly assessed.

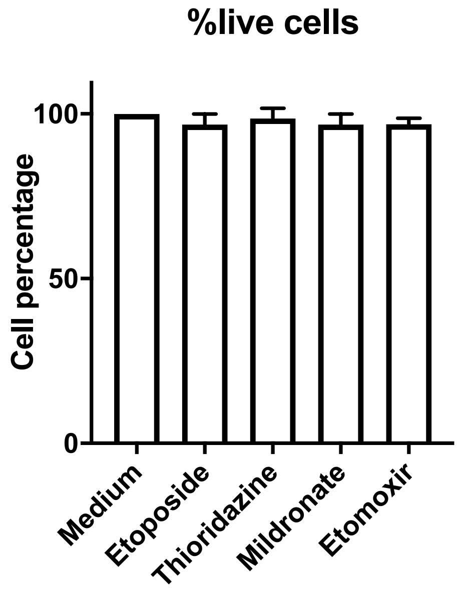

Supplementary Figure 2. Percentage of live cells. FACS analysis expressed as percentage of live cells related to untreated PBMCs exposed to etoposide, thioridazine, mildronate and etomoxir for 24 hours. All data represents mean ±SEM.
